# Biomaterials for Regenerative Cranioplasty: Current State of Clinical Application and Future Challenges

**DOI:** 10.3390/jfb15040084

**Published:** 2024-03-28

**Authors:** Lizhe He

**Affiliations:** Key Laboratory of 3D Printing Process and Equipment of Zhejiang Province, School of Mechanical Engineering, Zhejiang University, Hangzhou 310028, China; helizhe9203@zju.edu.cn

**Keywords:** cranioplasty, cranial defects, biomaterials, regenerative medicine, clinical trials

## Abstract

Acquired cranial defects are a prevalent condition in neurosurgery and call for cranioplasty, where the missing or defective cranium is replaced by an implant. Nevertheless, the biomaterials in current clinical applications are hardly exempt from long-term safety and comfort concerns. An appealing solution is regenerative cranioplasty, where biomaterials with/without cells and bioactive molecules are applied to induce the regeneration of the cranium and ultimately repair the cranial defects. This review examines the current state of research, development, and translational application of regenerative cranioplasty biomaterials and discusses the efforts required in future research. The first section briefly introduced the regenerative capacity of the cranium, including the spontaneous bone regeneration bioactivities and the presence of pluripotent skeletal stem cells in the cranial suture. Then, three major types of biomaterials for regenerative cranioplasty, namely the calcium phosphate/titanium (CaP/Ti) composites, mineralised collagen, and 3D-printed polycaprolactone (PCL) composites, are reviewed for their composition, material properties, and findings from clinical trials. The third part discusses perspectives on future research and development of regenerative cranioplasty biomaterials, with a considerable portion based on issues identified in clinical trials. This review aims to facilitate the development of biomaterials that ultimately contribute to a safer and more effective healing of cranial defects.

## 1. Introduction

Severe trauma and planned craniectomies commonly lead to acquired cranial defects, where parts of the cranial bone are fractured and/or removed from their original position [1]. The absence of cranium results in the sinking of the scalp and compromised cosmesis of the patient, while the loss of structural integrity of the cranium is clinically significant to cause disturbance of intracranial pressure and cerebrospinal fluid flow, resulting in deterioration of neurological function and motor ability [2,3]. These issues are clinically treated by cranioplasty, a surgical approach to reconstruct the skull and restore the normal shape, integral structure, and, more importantly, the original biological function of the cranium [4].

The essence of cranioplasty is to replace the defective cranial bones with cranial implants, which support the scalp to enhance cosmesis and instantly restore the flow dynamics of cerebrospinal fluid [5]. The gold-standard material for cranioplasty has long been considered an autologous cranium of the patient owing to its capacity for bone integration, mild immune responses, excellent anatomic fitting with the defective area, and good cosmesis outcome [6,7]. Nonetheless, autologous cranium grafts are limited and may suffer from microbial colonisation and loss of viable cells during the preservation period, causing post-operative graft resorption and infection [7,8,9]. To this end, synthetic materials such as polymethyl methacrylate (PMMA), calcium phosphate (CaP) ceramics, titanium plates/meshes, and polyether ether ketone (PEEK) plates were subsequently developed and applied to permanently replace the defected cranium [1]. Nonetheless, none of these materials are completely free from issues related to safety and comfort after implantation. For PMMA, the heat generated during in situ polymerisation and the residual monomers are known to jeopardise the viability of cells [10], whereas air bubbles trapped during setting increase the infection rate [11]; CaP, though known for its bioactivity allowing bone-bonding, is highly fragile and prone to fracture [12]. While titanium mesh is lightweight with adequate mechanical strength, the high thermal conductivity leads to scalp paraesthesia as environmental temperature changes, while other disadvantages include the potential for scalp thinning and artefacts in MRI imaging [13,14,15]. As for PEEK, a lack of biological integration with surrounding tissue may lead to implant loosening and strong foreign body reactions [16,17]. Besides the abovementioned drawbacks, most of these materials fail to properly interact with surrounding soft tissues, i.e., the scalp and temporalis muscle. For instance, the soft tissue coverage typically failed due to dehiscence at the implant–soft tissue interface as well as the colonisation of microbes, leading to disappointing cranioplasty [18,19,20]. There remains an imminent need to develop materials that can effectively repair cranial defects without raising safety concerns, causing discomfort, introducing artefacts in post-operative imaging, and interacting with the surrounding soft tissue in a desired way.

The advancement of tissue engineering and regenerative medicine, where biomaterials, living cells, and bioactive molecules modulating the biological responses of cells and tissues are integrated to induce the regeneration of specific tissues [21], has opened up possibilities for regenerative cranioplasty. Instead of being chemically inert and non-degradable to permanently fill the defect, a tissue-engineered biomaterial aims to stimulate the regeneration of a specified tissue coupled with implant resorption and ultimately heal the defect with completely regenerated tissue [22]. Regarding cranioplasty, a tissue-engineered construct is anticipated to induce complete cranium defect repair without material residuals, thus avoiding the long-term complications associated with the permanent presence of the implant as a foreign body material. Additionally, tissue engineering constructs are also promising to induce the regeneration of soft tissue (such as the scalp) to ensure healthy coverage over the defective sites [23,24]. Over the past decades, significant progress has been made in the research and development of materials for regenerative cranioplasty. Specifically, three types of biomaterials have been developed and successfully gone through clinical translation, with early-stage observation noticing their effects on stimulating the growth of native cranium [25,26,27]. Nonetheless, the long-term follow-ups reveal concerns over the safety of implants and call for solutions. To the best of our knowledge, no literature review focuses on the current state of regenerative cranioplasty implants regarding their material properties, the progress of clinical translation, or how future research will focus on improving the safety and effectiveness of these implants.

This narrative review focuses on recent advancements in biomaterials intended for regenerative cranioplasty. To demonstrate the regenerative capacity of the cranium, the first part of the work introduced the theoretical basis and clinical observation of spontaneous ossification after cranial defects and encouraged tissue engineering approaches to be applied in clinical treatment. Next, the work introduced three types of regenerative cranioplasty implants, namely, CaP/Ti composites, mineralised collagen, and 3D-printed PCL and composites thereof, for their composition, material properties, the progress of clinical translation, and the latest findings from clinical trials. Based on these contents, the third part discusses what potential future research will focus on, emphasising the need to address known issues identified in clinical trials and the theoretical understanding of cranial regeneration. This review aims to stimulate interest among biomaterial researchers and clinicians and provide insights for the development of cranioplasty implants that hopefully contribute to a safer and more effective healing of cranial defects.

## 2. Regeneration Capacity of Cranium

### 2.1. Biological Basis of Cranium Regeneration

While the idea of healing a cranial defect with bones regenerated in situ is attractive, it is reported that cranial defects in humans cannot spontaneously heal when the patient is older than 2 years old, with numerous factors considered to be involved [28]. The calvarial suture, where cells of osteogenic potential reside, is known to undergo a substantial functional closure around the age of 2 [29]. Additionally, the presence of type H capillaries, which are responsible for delivering cytokines that promote bone formation, diminishes in bone tissue as age increases [30]. The impaired potential for tissue regeneration along with ageing is also accompanied by senescence of cells, which leads to a reduced number of osteoblasts and downregulation of genes related to osteogenic differentiation (e.g., regulatory and downstream genes in the Runx2 signalling pathway) [31,32,33,34]. It is even proposed that the loss of regeneration capacity is related to a loss of stem cells with chondrogenic capacity as age increases, even though calvaria predominantly develops through intramembranous ossification and chondrogenic activities are less involved [35].

**Figure 1:**
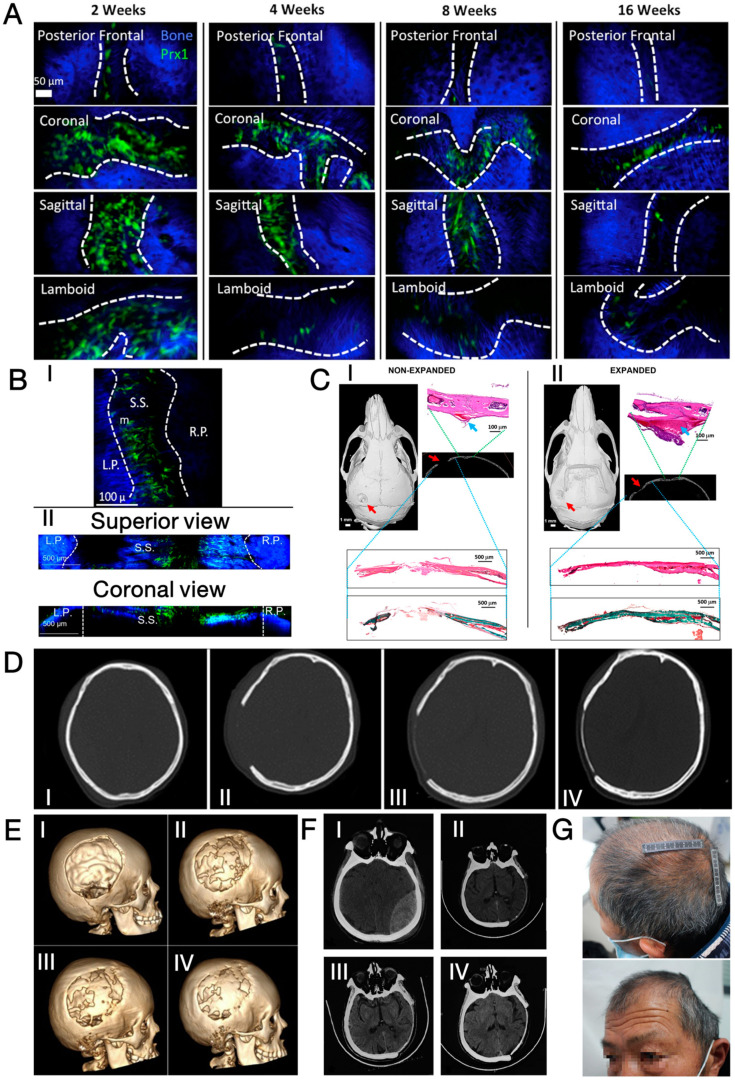
(**A**) Spatial distribution of Prx1+ cells in calvarial sutures of PRX1-creER-EGFP+/− mice at different ages. Dashes indicate the border of calvarial sutures [39]; (**B**) spatial distribution of Prx1+ cells in sagittal sutures of PRX1-creER-EGFP+/− adult mice (2-month-old) (I) before and (II) 7 days after mechanical expansion. Dash lines demarcate the rims of sagittal suture (S.S.) that separates left parietal bone (L.P.) and right parietal bone (R.P.) Dashes indicate the border of calvarial sutures [29]; (**C**) micro-CT image, 3D reconstruction model, and histological sections of sagittal suture (green dashed lines, with magnified view) and cranial defects (blue dashed lines, with magnified view) 60 days after craniectomy in control and suture expansion groups, showing increased number of Prx1+ cells in expanded suture [29]; (**D**) CT images of a patient’s (female, 20 years old) cranium taken pre-operatively (I), POD 3 (II), POW 7 (III), and POW 23 (IV), showing spontaneous cranial regeneration [47]; (**E**) three-dimensional reconstruction of a patient’s (female, 7 years old) cranium at POD 0 (I), POM 10 (II), POM 16 (III), and POM 26 (IV), showing progressive cranial regeneration [48]; (**F**) CT images of a patient’s (male, 64 years old) cranium taken pre-operatively (I), POD 145 (II), POD 171 (III), and POM 23 (IV), showing progressive cranial regeneration [49]; (**G**) photographs of the 64-year-old patient taken at POM 23 [49]. Reprinted from Refs. [29,39,47,49]. Reprinted with permission from Ref. [48]. Copyright 2024, Elsevier, Amsterdam, The Netherlands.

Nevertheless, as reported by Alleyne et al., the genetic expression of bone morphogenic protein-2 (BMP-2), BMP-7, and transforming growth factor-β1 (TGF-β1) in adult rats was all slightly upregulated within 1 week of craniectomy, indicating an innate mechanism attempting to repair the cranial defects via bone regeneration [36]. Considering that the cranial suture contains osteogenic fronts where cranium growth actively takes place, researchers focused on the cranial suture for its potential role in cranial defect regeneration. In 2014, Ouyang et al. targeted cells expressing paired-related homeobox gene-1 (Prx1/Prrx1) in the cranial suture of mice and confirmed the cells to have the potential for both osteogenic and chondrogenic differentiation [37]. Meanwhile, Zhao et al. identified Gli1-expressing cells in the suture mesenchyme of mice; see Figure 1A [38]. Upon the occurrence of cranial defects, these cells showed an ability to migrate towards the defect centre to facilitate tissue regeneration [39,40]. More recently, Prx1+ cells were “engineered” to induce cranial defect regeneration. Aldawood et al. applied mechanical force to expand the sagittal suture in skeletally matured young adult mice (2 months old) [29]. As shown in Figure 1B, the sutural expansion resulted in the enhanced proliferation of Prx1+ cells inside the suture mesenchyme without altering the genetic expression profile when compared to cells in a naturally expanding cranial suture. Following the creation of a critically sized cranial defect near the expanded sagittal suture, near-complete bone regeneration was observed 60 days later (Figure 1C). However, the suture expansion process failed to induce cranium regeneration in older (10-month-old) mice, indicating limited cranial regeneration capacity in aged animals.

In the meantime, researchers also refer to developmental biology for inspiration in stimulating the regeneration of the cranium. It is believed that frontal, temporal, and occipital bones originate from cells derived from the neural crest and cephalic mesoderm [41] and ossify via intramembranous ossification, where bones are directly formed without transition from cartilages: sprouting vessels originated from existing vasculature enter the non-ossified mesenchyme condensation and transport proteins, mineral ions, and nutrients, leading to ossification taking place at the centre of mesenchyme condensation near the blood vessels, accompanied by outward migration of mesenchyme cells to expand the mesenchyme condensation. These activities continue until mesenchymal cells receive certain signals to stop migration, and the fusion of previous ossification sites gives rise to mature bone tissue [42]. According to animal models, the intramembranous ossification process is predominantly involved in the spontaneous regeneration of holes drilled into bones, including calvaria, possibly owing to a lack of mechanical motion at the defect sites [43].

An upregulated expression of Runx2 and osterix (osteogenesis-related) rather than Sox9 (chondrogenic-related) is vital to the intramembranous ossification process [44]. As reflected in the post-trauma gene expression profile, the BMP/Smad signalling pathway is actively involved as an upstream modulator of osteogenic gene expression [36]. The Wnt signalling pathway, which suppresses Sox9 expression (in the absence of fibroblast growth factor) and subsequent chondrogenic differentiation of mesenchymal stem cells, is also considered involved in the intramembranous ossification pathway [45]. Readers are referred to works by Ko and Sumner [43] and Chen et al. [46] for a detailed discussion of signalling pathways and key molecules involved during intramembranous bone regeneration and cranial defect healing, respectively.

### 2.2. Clinical Observation of Cranium Regeneration

While more studies are currently underway to explore potential mechanisms and novel strategies for inducing spontaneous cranial regeneration, there have been a growing number of publications in recent years reporting unexpected spontaneous regeneration of the cranium in patients older than 2 years. These publications were initially sourced through a literature search of the Web of Science database using the query “TS = (craniectomy OR craniectomies OR cranioplasty OR cranial OR cranium) AND TS = (regenerate OR spontaneous OR ossification OR bone formation)”. Subsequently, the results were carefully examined to identify publications documenting cases of cranial defect repair that occurred spontaneously, without the involvement of tissue engineering constructs or biological stimuli, such as platelet-rich plasma, stem/progenitor cells, cytokines, stromal vascular fraction, and any drugs known to upregulate osteogenesis.

The included cases were summarised in Table 1, and Figure 1D–G showed evidence of cranium defect healing confirmed by medical imaging. The ages of patients in these cases were mostly below 30 years, with the oldest patient being 64 years old at the time of the report (see Figure 1F,G for signs of cranium regeneration in this case). Although there are differences in the surgical procedures, many of these reports claim to meticulously treat the tissues at the defective sites. This included either not opening the dura mater or sealing/reconstructing it with synthetic dural patches, saline irrigation to cool down the craniectomy field to protect the brain and cranium, and preserving the vascularisation of the pericranial flap. Consequently, the tissues were believed to preserve viable cells to the maximum extent possible, while irritation to the native vascularisation was kept to a minimum. Generally, spontaneous regeneration of the cranium was first observed 2–3 months after surgery as isolated, island-like bone tissue over the dura mater, with progressive ossification achieving near-complete cranium regeneration as early as 2 years post-operatively.

To sum up, the conventional understanding that the cranium is unable to regenerate has been challenged in recent years. On the one hand, studies in developmental biology have revealed the presence of stem cells residing in cranial sutures that are capable of self-renewal and osteogenesis, as well as key molecules and important signalling pathways involved. On the other hand, cases of spontaneous cranial regeneration, though currently regarded as serendipitous, are increasingly being reported. It is reasonable to believe that with the application of tissue engineering constructs, a full-thickness, full-sized regeneration of the cranium will be achieved to heal cranial defects. To this end, three types of regenerative cranioplasty implants have been developed and found to be useful, as discussed in the next section.

**Table 1:** Cases of spontaneous ossification/regeneration of cranium after craniectomy.

Age (Years)	Gender	Indications of Craniectomy	Highlights of the Surgical Process	Post-Operative Outcome	Ref.
6	Male	TBI	Pericranium was gently and carefully dissected to preserve the blood vessels. Bifrontal craniectomy was performed to alleviate intracranial pressure. The Dura mater was opened, followed by closure and duroplasty with a synthetic dural patch (Neuro-Patch^®^, B Braun, Tuttlingen, Germany). The pericranial flap was placed over the Dura mater before final closure.	POD 110: both frontal lobes were covered and surrounded with an abnormally hyperdense layer. Eggshell-shaped bony islands were distributed within the craniectomy area and were proven to be bone tissues via pathological examination.	[50]
7	Female	TBI	A craniectomy was performed (~12 cm) and an open complex cranium fracture was treated. Dura mater lacerations were treated with a synthetic dural patch (Durepair™, Medtronic, Minneapolis, MN, USA)	POM 10: isolated islands of regenerated bones presented in the craniectomy area POM 16 and 26: continuous bone reaching a near-complete cranial regeneration in the craniectomy area	[48]
8	Female	Brain abscess	Craniectomy was performed (~7 cm) to treat the brain abscess. The Dura mater was opened during surgery, followed by primary dural closure without duroplasty.	POY 2: near-complete regeneration of cranium in the craniectomy area, with only a few areas of void.	[51]
12	Female	Tumour	Craniectomy performed for radical excision of a medulloblastoma	POY 2: extensive new bone formation almost completely covering the earlier craniectomy area.	[52]
15	Male	TBI	Craniectomy was performed (~7 cm) to treat the extradural hematoma. The Dura mater was opened during surgery, followed by duroplasty using autologous pericranium (harvested as a pedicled flap).	POM 3: new bone formation as separate islands in the craniectomy area POM 6: the cranium underwent significant reconstruction spontaneously, achieving a contour comparable to the native cranium before cranioplasty	
18	Male	TBI	Pericranium was reflected with a scalp flap. A bilateral craniectomy was performed to alleviate intracranial pressure. Dura mater was opened on left and ride sides, followed by duroplasty with synthetic dural patches (left: DuraGen^®^, Integra, Princeton, NJ, USA; right: Dura-Guard^®^, Synovis, St. Paul, MN, USA).	POD 33: early heterotopic ossification only on the right side POD 107: both sides showed heterotopic ossification POD 160: significant recovery of the cranium, with thick bone pieces presented along the ridges on the right side and eggshell-like thin bones along the dura on the left side	[53]
20	Female	TBI	The whole scalp flap (including pericranium) was elevated as an integral part. Craniectomy (10 ∗ 10 cm) was performed to treat an extradural hematoma. Dura mater was unopened. Pericranium and subcutaneous tissue were closed using absorbable sutures as the inner layer.	POW 7: ossified and remodelled bone distributed within the craniectomy area and attached firmly to the dura mater. POW 23: regenerated cranial bone spread nearly throughout the craniectomy site, which was solid and uninterrupted.	[47]
29	Female	TBI	Craniectomy (~10 cm) was performed to treat the subdural hematoma and brain oedema. Saline irrigation to avoid high temperature during craniectomy to protect the dipolë. The temporal muscle and pericranium were dissected in a single plane to respect the pericranium. The Dura mater was opened, and a synthetic dural patch (DuraGen^®^, Integra, Princeton, NJ, USA) was placed between the dural edges for reconstruction.	POY 2: thin bone regenerated throughout the craniectomy area	[54]
64	Male	TBI	Craniectomy (8 × 10 cm) was performed to treat an extradural hematoma. Dura mater was unopened. The cranial capsule, dura mater, and gelatin sponge were sealed with absorbable sutures as the inner layer. Skin, subcutaneous tissue, and gelatin sponge were sealed with non-absorbable sutures as the outer layer.	POD 145: regenerated bone islands distributed within the craniectomy area. POD 171: bone islands expanded and fused, leading to near-complete coverage of the craniectomy area. POM 23: complete regeneration of hard cranium, but significantly thinner than the contralateral side.	[49]

Abbreviations: TBI = traumatic brain injury, POD/POW/POM/POY = post-operative days/weeks/months/years.

## 3. Three types of Regenerative Cranioplasty Implants and Progress of Clinical Translation

This section introduces three types of regenerative cranioplasty implants, namely the calcium phosphate/titanium (CaP/Ti) composites, mineralised collagen, and 3D-printed polycaprolactone (PCL) and its composites. Following a series of studies to improve safety and effectiveness, all these materials have received clinical translation in the past decade. While many of the studies reported exciting results with evidence of guided cranium regeneration, the incidence of post-surgical complications was also documented, raising concerns among clinicians and calling for solutions derived from biomaterial research. This section aims to highlight the advantages of each type of material as well as their known limitations that require further effort to resolve.

A search of available literature was performed in the Web of Science database using the query “TS = (cranium OR cranial OR calvarial OR cranioplasty OR calvaria) AND TS = (regenerate OR tissue engineering OR tissue engineered OR regenerative medicine OR regeneration).” Research (journal) articles and communications reporting clinical follow-up were then carefully subjected to the second round of screening, with the publications reporting the use of (partly) bioresorbable, commercialized biomaterials intended for neurosurgical applications included for discussion. Works cited in these publications reporting the methodologies for material synthesis and manufacturing, characterisation of material properties, and (pre-) clinical studies were also included for discussion.

### 3.1. CaP/Ti Composites

The composites of CaP and titanium typically consist of two phases in a heterogeneous combination. The backbone is a lightweight, macro-porous framework of titanium. The excellent mechanical properties of titanium provide strength, stiffness, and toughness to the entire structure, granting the implant energy absorption capacity while remaining moderately malleable to conform to the actual shape of cranial defects [1,55]. CaP, known for its high fragility but excellent bone-bonding (osteoconductive) bioactivity and allowance of bone tissue ingrowth into its porous structure, fills the voids of the porous titanium backbone to biologically integrate with surrounding tissues [1,12,56]. Such a combination harnesses the advantages of both components, resulting in a mechanically robust, moderately malleable, and biologically active cranioplasty implant [57].

**Figure 2:**
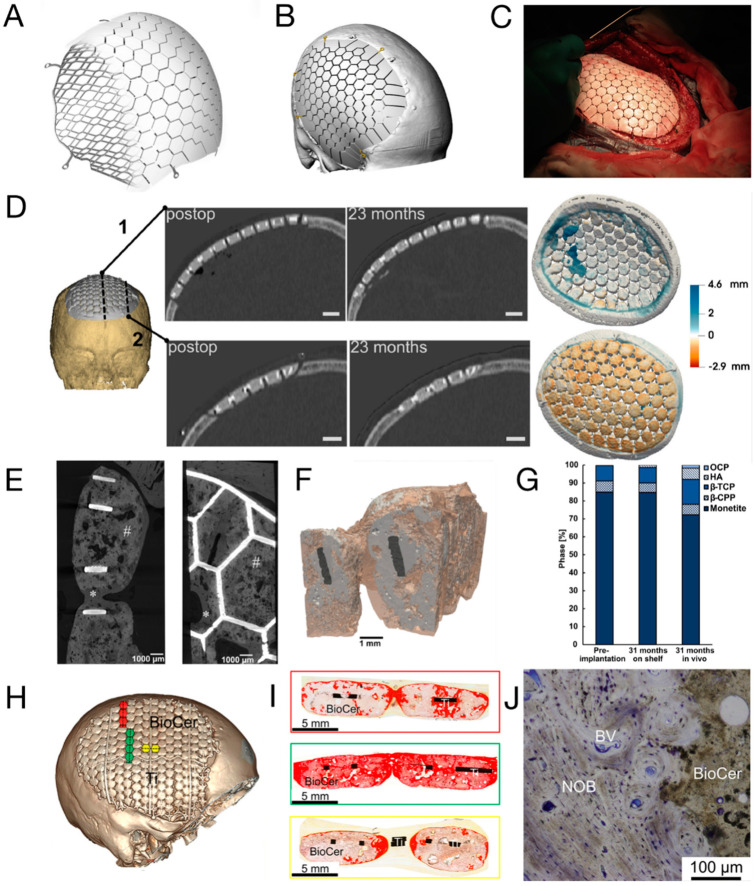
(**A**,**B**) Demonstration of OssDsign^®^ PSI showing the titanium backbone covered by CaP (BioCer) tiles and a schematic view after implantation [60]; (**C**) photographs showing the inlay implantation of an OssDsign^®^ PSI [60]; (**D**) CT images of two planes and the corresponding 3D reconstruction from a clinical case. Resorption of CaP tiles occurred mainly on the pericranium/scalp side, while new bone formation was evident on the dural side [63]. (**E**,**F**) CT images show a honeycomb-like titanium framework (white in E, black areas in F), as well as new bone (* in (**E**), pale brown in (**F**)) formation bridging and over the CaP tiles (# in (**E**), grey areas in (**F**)) 31 months post-operatively [63]. (**G**) Compositional change in mineral phases of CaP tiles before and after 31 months on a shelf or in vivo, demonstrating the formation of hydroxyapatite (HA) [63]; (**H,I**) histological staining of new bone (red) formation in three regions of the cranioplasty implant retrieved at POM 21. Black parts are titanium backbone of the implant [25]. (**J**) Toluidine blue staining of an implant retrieved 21 months post-operatively shows the deposition of new bone (NOB)-containing blood vessels (BVs) over the surface of CaP (BioCer) [25]. Reprinted from Refs. [25,60,63].

A representative CaP/Ti composite that is commercialised and subjected to clinical trials is the OssDsign^®^ cranial patient-specific implants (OssDsign^®^ PSI, Uppsala, Sweden). As shown in Figure 2A, the lightweight titanium backbone is customised based on the actual skull defect and additively manufactured to yield a patient-specific size and curvature. This facilitates the inlay implantation process and achieves optimal cosmesis while minimising artefacts in X-ray imaging (see Figure 2B,C) [58,59]. In the latest design, fixation arms are integrated into the titanium mesh to ease intraoperative fixation (see Figure 2A) [60]. The other part of the implant is a formulated CaP, moulded onto the titanium mesh to create solid tiles with a thickness of 6 mm [25]. An inter-tile gap of around 1 mm further allows the perfusion of liquid across the implant to facilitate the healing process [25]. With a porosity of approximately 43%, constituted by macropores with a diameter of around 600 μm [25], the CaP tiles can also absorb antibiotics (e.g., gentamicin [61] and vancomycin [62]) and later release the antibiotics to prevent surgical site infection.

A distinctive feature of the OssDsign^®^ implant is that the dissolution of its formulated CaP tiles is nearly synchronized with new bone formation, ultimately leading to the replacement of CaP by a regenerated cranium [62]. The CaP is a compound consisting of monetite (~86%), β-tricalcium phosphate (~8%), β-calcium pyrophosphate (~6%), and brushite [63]. As the major phase, monetite is known for its intermediate dissolution rate between rapid-degrading brushite and the hardly soluble hydroxyapatite, as well as its ability to induce ectopic bone formation [64,65]. Meanwhile, the presence of β-calcium pyrophosphate critically delays the degradation of the CaP compound and stimulates osteogenic activities [63,66]. In early clinical case studies, new bone formation was observed within the implant after ≤30 months of implantation (see Figure 2D) [67]. High expression of genes related to osteogenic activities was seen in the central region of the implant, while histology examination revealed vascularized bone formation adjacent to and within the porous CaP tiles throughout the implant [61,68]. A closer examination of the tissue responses and the degradation of CaP was later performed. Based on the sheep cranioplasty model, remodelling of the periosteum and endosteum was seen on the superior and inferior sides of the CaP tiles, whereas the pure titanium mesh was encapsulated by thick fibrotic tissue. Conversion of CaP into bone-like carbonated apatite (e.g., hydroxyapatite) was evident at the proximity (<100 um) of the CaP tiles, and the trend was more pronounced at the implant periphery (the implant-remnant cranium interface) compared to the central region, as highlighted in Figure 2E–I. Meanwhile, the presence of macrophages and osteoclasts at the CaP surface indicated cell-mediated resorption of the ceramic tiles, while the presence of blood vessels embedded in the bone matrix demonstrated the ongoing remodelling of the newly formed cranium; see Figure 2J [25]. While the declining volume (especially in the central region) of the implant by <20% after 3 years of implantation in the human cranium suggested the degradation of the ceramic phase, the increased apparent density throughout the whole implant demonstrated concurrent new bone formation in the meantime [63]. Together, these data suggest that bone formation and remodelling are coupled with CaP degradation at a similar rate.

Recent years have seen the publication of single-centred retrospective studies on cranioplasty using OssDsign^®^ implants. Linder et al. presented the results of 50 patients who underwent cranioplasties [60]. With a median follow-up period of 25 months, only 1 and 2 patients developed early post-operative infection and wound dehiscence, respectively. Considering that 64% of the patients involved had experienced failed cranioplasties previously, the outcome indicated the promising effectiveness of the novel implant. In 2022, Sorek et al. reviewed the outcome of the largest single-centre study in America [58]. Among the 18 patients included in the study, the cosmetic outcome was satisfying, and a minor improvement in neurological function was reported. Although one patient died and three patients reported perioperative complications, none of these cases were directly related to the implants, indicating an acceptable safety profile of the implant. It is assumed that the vascularization over and within the implants helped reduce the infection rate. The implants were also applied in paediatric cranioplasty [69]. Within the follow-up period, with a median of 15 months, no post-operative infection was reported among the six children.

Comparisons between OssDsign^®^ implants and mainstream cranioplasty options, such as autologous bone, titanium, PMMA, and PEEK, have been extensively studied. A study by Koller et al. found that the outcomes of cranioplasty with OssDsign^®^ were comparable to those achieved with implants currently in clinical use. Additionally, the study suggested potential advantages of OssDsign^®^, including a shorter operation time and a lower post-operative infection rate, although statistical significance was not reached [70]. More recently, Wesp et al. investigated the complication rate after cranioplasties with either PMMA or OssDsign^®^ implants [71]. A total of 13 post-operative complications occurred in patients receiving PMMA implants, whereas only 6 patients implanted with OssDsign^®^ were found to have complications, despite a lack of statistical significance. However, the rate of implant removal (9 PMMA patients vs. 1 OssDsign^®^ patient) and surgical site infection (6 PMMA patients vs. 0 OssDsign^®^ patient) were both statistically higher for those implanted with PMMA. The authors also noted that the retrieved OssDsign^®^ implant was considerably integrated with the surrounding cranium and enveloped by a vastly vascularized layer, in contrast to PMMA implants that were loosely linked to the tissues nearby. To date, a total of 1995 cranioplasties using OssDsign^®^ implants were performed, and none of the 66 explantation cases were a consequence of the implant-induced failure, according to the latest post-marketing surveillance review [72]. Nonetheless, it shall be noted that the titanium backbone is non-degradable and is expected to be permanently placed at the implantation site. The presence of titanium may impair the quality of magnetic resonance imaging for diagnosis [15]. In addition, it shall be noted that the Young’s modulus (~110 GPa) of titanium is much higher than that of human cortical bone (~20 GPa), so the newly formed bone undergoes stress shielding and, eventually, is prone to fracture [73]. A longer follow-up is also required to demonstrate whether the presence of residual titanium backbone causes any safety concerns.

### 3.2. Mineralised Collagen

**Figure 3:**
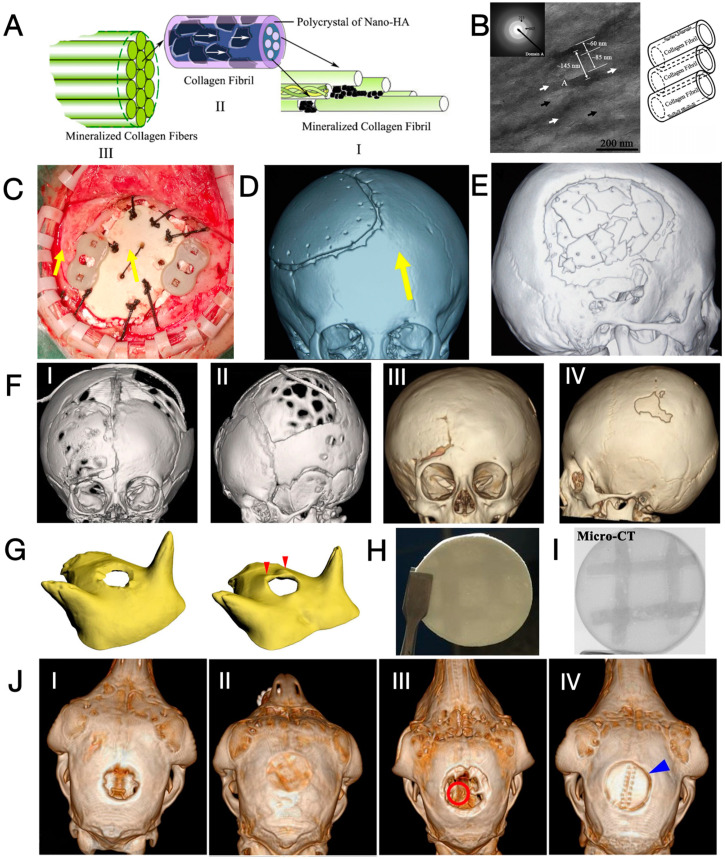
(**A**) Scheme of the hierarchical structure of MC with biomimicry to bone matrix nanostructure [76]; (**B**) scheme, transmitted electron microscopic image and selected area diffraction pattern of MC. Black arrow: collagen fibril; white arrow: mineral crystals in parallel direction. Insert picture shows the selected area diffraction pattern of domain A [76]; (**C**) photographs of an implanted MC fixed by PEEK plates (between yellow arrows) and 10/0 threads (in black) [85]; (**D**,**E**) CT images show an enlarged gap between MC and remnant cranium at POY 1, and a fragmented MC at POY 2, and yellow arrows highlightes the border of resorbed implant [85]. (**F**) Pre-operative CT images of a 4-month-old patient with a cranial defect (I, II) and post-operative CT images taken at POY 3 after reconstruction using ReFit^®^ MC (III, IV) [88]. (**G**) Cranium of baby sheep after cranioplasty with compact MC/PCL (left, uneventful) and titanium mesh (right, with cranial protrusion marked by red arrows) [91]. (**H**,**I**) Photographs and micro-CT images of hybrid MC/PCL composites [90]; (**J**) Post-operative CT reconstruction of the cranium of baby sheep at POM 3, treated with blank (I, minimal regeneration), hybrid MC/PCL (II, complete regeneration), porous MC/PCL (III, partial regeneration with remaining voids highlighted with red circle), and compact MC/PCL (IV, minimal degradation without bone-implant bonding at the interface, marked by blue arrow) [90]. Reprinted from Refs. [85,90]; Reprinted with permission from Refs. [76,88]. Copyright 2024, Elsevier; Ref. [91]. Copyright 2017, American Chemical Society.

The need for materials that recapitulate the native cranium while possessing biodegradability has driven researchers to create synthetic bone-like materials. These materials aim to replicate not only the composition but also the micro- and nanostructure of native bone. One such synthetic bone-like material is mineralised collagen (MC), which consists of collagen—the primary organic component of native bones—and hydroxyapatite, which shares a chemical composition similar to the apatite in bone mineral [74,75]. Moreover, the nanostructure of MC mirrors that of native bones through biomimicry: platelet-like hydroxyapatite crystals are arranged in parallel with the long axis of collagen fibrils, both on the surface and within the intrafibrillar spaces among collagen fibrils (see Figure 3A,B) [76].

MC with a bone-like nanostructure is typically prepared through a self-assembly process [77,78]. For instance, collagen is first dissolved in a dilute aqueous solution of acid (e.g., acetic acid or hydrochloric acid). Next, two solutions rich in calcium (e.g., CaCl_2_) and phosphorous (e.g., H_3_PO_4_ or NaH_2_PO_4_) are slowly added into the collagen solution and homogenized, with the Ca/P ratio adjusted to 1.67 to facilitate the hydroxyapatite precipitation. Then, the pH of the mixture is adjusted to 7.4 or 9, followed by ageing, centrifuging, washing, and freeze-drying to obtain the MC. Alternatively, Kikuchi et al. proposed an MC preparation process by slowly adding collagen/H_3_PO_4_ solution and Ca(OH)_2_ suspension into water [79].

The clinical application of MC has been underway in the past decade, with cases reporting successful treatment of cervical interbody fusion [80], non-union long bone fractures [81], and calcaneal fractures [82]. An early attempt at neurosurgical application was reported by Qiu et al., where three patients with craniotomy burr-holes were reconstructed with MC plugs [83]. Within the follow-up period of up to 12 months, all surgeries were successful without post-operative inflammation, itching, or exudation. Radiographs showed a successful fusion of the MC and surrounding cranium. Meanwhile, the bone mineral density within the defects increased and was close to that of the native cranium, demonstrating bone regeneration at the implant site.

The biodegradability and osteogenic potential have piqued the interest of physicians, leading to the application of MC in paediatric cranioplasty, as the patient’s cranium and bone defect undergo dynamic development and should not be constrained by rigid, non-degradable implants [5,84]. In 2014, a single-centre clinical trial investigating the effectiveness of MC in paediatric cranioplasty was initiated. The one-year follow-up of 13 children revealed generally uneventful healing and enhanced cognitive function [26]. Among the cases, one experienced post-operative subcutaneous hydrops due to an epidural defect, while another reported implant cracking without compromising stability. An update in 2018 from the same group reported on a larger cohort (45 cases) with follow-up periods ranging from 8 months to 3 years [85]. All surgeries were successfully performed, yet they reported 14 cases of subcutaneous hydrops, 4 cases of implant loosening (Figure 3D), 1 case of wound indolence, and 1 case of complete implant fragmentation (Figure 3E), accounting for 17 patients in the entire cohort. To avoid these complications, the authors proposed good post-operative hygiene practices and enhancement of operative skills, including the use of PEEK fixation plates, proper handling of the temporalis muscle, prompt repair of the dural defect, and tight suspension of the dura mater. In a more recent retrospective review, 65 cases with a minimum follow-up period of 2 years were investigated. A total of 42 cases of complications were reported, including 27 cases of subcutaneous hydrops, 8 cases of implant loosening (Figure 3D), 6 cases of implant fragmentation, and 1 case of sterile inflammation [86].

Another mineralised MC product utilised in paediatric cranioplasty is marketed as ReFit^®^ (Hoya Technosurgical Co., Tokyo, Japan). This porous material boasts an overall porosity of 35% and a pore size ranging from 100 to 500 μm, imparting sponge-like elasticity and flexibility [87]. In an early case report, autologous bone grafts were harvested bilaterally from the cranium of two children (6 and 11 years old), leaving two cranial defects that were repaired with autologous bone dust and ReFit^®^, respectively. A post-operative CT scan conducted after 1 year revealed a larger area of bone formation in the ReFit^®^ group. However, it is noteworthy that the thickness of the regenerated cranium was less than 50% of the original nearby cranium [87]. Later, this material found application in the cranium reconstruction of a 4-month-old female, leading to significant calcified tissue generation three years post-operatively without any reported complications (see Figure 3F) [88].

In addition to standalone applications, recent studies have focused on utilising mineralised collagen (MC) as a bioactive agent in polycaprolactone (PCL)-based composites, which exhibit either compact or porous structures. In these studies, MC demonstrated superior performance compared to titanium, polymethylmethacrylate (PMMA), and hydroxyapatite/PCL composites in upregulating the osteogenic differentiation of mesenchymal stem cells [89,90]. While a compact MC/PCL composite exhibited commendable compressive strength (0.59 ± 0.01 kPa vs. 0.015 ± 0.001 kPa) and elastic modulus (32.63 ± 0.75 MPa vs. 1.06 ± 0.03 MPa) in comparison to porous composites, the latter with a pore size of 20–150 μm and a porosity of 73.6 ± 2% facilitated bone tissue ingrowth into the pores. This contrasted with the minimal degradation observed in the compact counterparts when tested in the cranial defects of 1-month-old sheep [91]. Defects repaired with titanium mesh or PMMA showed little bone formation. Furthermore, PMMA exhibited fragmentation due to its high brittleness, and the cranium beneath the titanium developed protrusions, possibly attributed to the excessive stiffness of titanium (see Figure 3G) [91]. The authors concluded that MC/PCL composites induced bone growth without premature failure or distorting the cranial shape due to moderate mechanical stiffness, and a hybrid structure with grid-like compact composites as reinforcement and porous composites as matrix (see Figure 3H,I) was later designed. The hybrid composite balanced the bone ingrowth capacity and mechanical properties of implants, outperforming purely porous MC/PCL composites to induce more efficient bone growth, whereas compact composites showed minimal degradation and a lack of bonding to the surrounding cranium (Figure 3J) [90].

To sum up, the MC developed in the early 2000s was predominately applied to paediatric cranioplasty. Despite having bone-mimetic composition and nanostructure, the single-centred retrospective studies reported high complication rates. A critical issue, as surgeons pointed out, is the slow bone growth at the cranium/implant interface that is outpaced by brain expansion, causing dislodgement and fragmentation of the cranium [85]. Meanwhile, the early fragmentation suggests an insufficient strength of the material [92], while the fast degradation nature of collagen may also exacerbate the loss of strength [93]. As collagen is a naturally sourced material, extra efforts must be made to remove all immunogenic and pathogenic impurities [94]. More research is required to couple brain expansion/cranial growth with implant degradation and new bone formation while enhancing the strength and toughness of MC.

### 3.3. Three-dimensional-Printed PCL and Its Composites

Synthetic biodegradable polymers have long been a preferred choice among researchers for developing biodegradable tissue engineering constructs, with one of the extensively studied materials being PCL [95]. Recognised for its excellent biocompatibility, slow degradation rate, and compatibility for both solvent- and melt-based processing, PCL has received approval from the Food and Drug Administration (FDA) for use in drug delivery devices and resorbable sutures [95]. As a thermoplastic polymer with a low melting point, PCL is fully compatible with fused deposition modelling (FDM), a 3D-printing process where molten polymers are incrementally added into the building volume, layer by layer, to fabricate a three-dimensional object [96]. The application of 3D printing allows for the fabrication of models with intricate geometries and porous structures in an automated manner, which is valuable for the production of customised cranioplasty implants tailored to the unique size and shape variations in each case of cranial defects [97].

A series of studies since the early 2000s has culminated in the development of 3D-printed, patient-specific, porous, fully biodegradable cranioplasty implants based on PCL and its composites. Early in 2003, it was reported that 3D-printed PCL, with a porosity of approximately 70% and seeded with either osteoblasts or mesenchymal stem cells, exhibited greater efficacy than cell-free scaffolds and a blank group in inducing regeneration at critically sized cranial defects [98,99]. Recognising the porous structure’s potential to load bioactive cues for facilitating bone regeneration, the team subsequently immersed the 3D-printed PCL in the autologous, heparinised bone marrow of pigs, followed by implantation into the pigs’ orbital wall defect. In comparison to blank or bone marrow-free counterparts, the loading of autologous bone marrow resulted in more effective bone regeneration (14.1% vs. 4.5% vs. 0%) [100].

**Figure 4:**
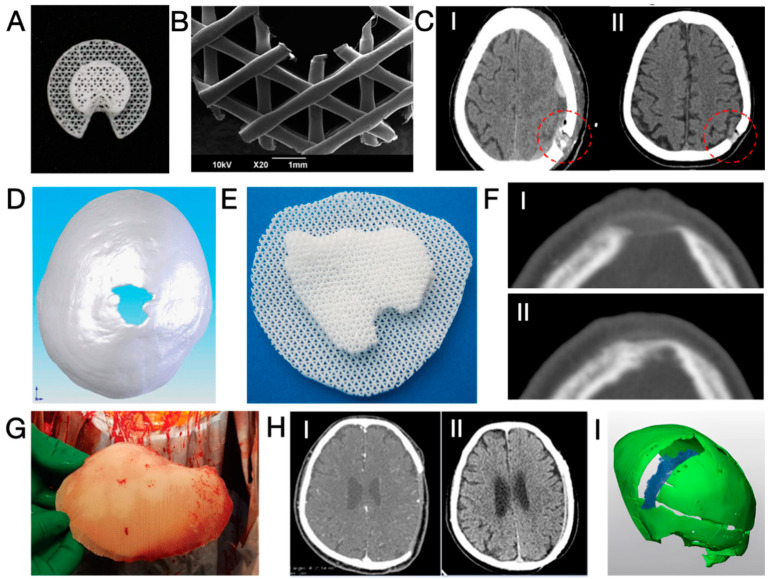
(**A**,**B**) Photographs and microscopic images of Osteoplug^®^ burr hole plug [104]; (**C**) CT images taken post-operatively (I) and at POM 15 (II), showing increased bone volume at the burr hole after implantation with a 3D-printed PCL plug. Red circles highlight the defected areas [103]; (**D**,**E**) a 3 × 3 cm^2^ cranial defect and the corresponding customised 3D-printed PCL cranioplasty implant [27]; (**F**) CT images of the defect shown in (**D**), taken post-operatively (I) and at POM 6 (II), showing bone regeneration [27]; (**G**) Photograph of a 3D-printed, customised PCL cranioplasty implant taken intraoperatively [106]; (**H**) CT images of a cranial defect (9.7 × 8.9 cm^2^) taken post-operatively (I) and at POM 20 (II), showing significant restoration of the cranium structure after reconstruction with a 3D-printed PCL implant [105]; (**I**) three-dimensional reconstruction of a band-shaped cranial defect at POM 12 after frontal-orbital advancement and cranioplasty using 3D-printed PCL mesh, showing a significant void surrounding the cranium (in green) and occupied by partly degraded PCL (in blue) [107]. Reprinted from Refs. [104,105,106]; Reprinted with permission from Ref. [103]. Copyright 2017, Future Medicine Ltd.; Ref. [27]. Copyright 2024, Georg Thieme Verlag KG; Ref. [107]. Copyright 2024, Wolters Kluwer Health, Inc., Philadelphia, PA, USA.

The first report on the neurosurgical application of 3D-printed PCL was published in 2006 [101]. In this study, five patients received 3D-printed PCL plugs (later commercialised as Osteoplug^®^, Osteopore, Singapore; see Figure 4A,B) to fill burr holes with a diameter of 14 mm. All plugs were coated with autologous blood and sealed with bone using fibrin glue. A 12-month follow-up revealed that all cases experienced uneventful recovery, achieving satisfying cosmesis. CT images indicated increasing bone formation since post-operative month (POM) 3 in the defective area of an 83-year-old patient. Similarly, another study reported 12 cases of uneventful burr hole reconstruction, observing simultaneous progressive bone regeneration [102]. Recent publications have noted that the use of PCL plugs resulted in effective bone regeneration after ~1 year (see Figure 4D) and significantly higher scores for both aesthetic outcomes and quality of life (QOL) without an increase in complication rates in comparison to patients with untreated burr holes who suffered from scalp depression [103,104].

The application of 3D-printed PCL in the reconstruction of larger cranial defects began in 2010 when Probst et al. repaired a 3 × 3 cm^2^ irregular cranial defect in an 11-year-old female; see Figure 4D–F [27]. To enhance bone-bonding bioactivity and stimulate bone growth, a PCL composite containing 20 wt.% of β-tricalcium phosphate (β-TCP) was utilised. CT images (Figure 4F) revealed signs of integration between the implant and the surrounding cranium six months after the surgery without any reported complications. Even larger implants (see Figure 4G) were successfully applied to induce cranium regeneration in later cases. In 2021, Hwang et al. reported two cases of cranioplasty using 3D-printed, patient-specific β-TCP/PCL in adults (21 and 25 years old), where the size of cranial defects exceeded 9 × 8 cm^2^ [105]. The implants underwent a 30 min immersion in bone marrow aspiration before being securely fixed into the defects. No complications were reported, and CT images (Figure 4H) indicated that the cranial defects were “completely filled” in both cases. The radiodensity within the defect after 8 months of implantation increased from 50 to 158 Hounsfield units (HU), demonstrating bone regeneration, although the radiodensity remained significantly lower than that of the native cranium (900–1000 HU). Koo et al. conducted a revision cranioplasty for a 73-year-old male using a 3D-printed PCL implant covered with a latissimus dorsi musculocutaneous flap [106]. After 1 year, a certain degree of bone regeneration and bone-implant integration was noticed.

Despite the previously reported uneventful healing, a recent study by Gonzales Matheus and Phua provides an alternative perspective [107]. The authors employed non-customised 3D-printed PCL meshes (without intraoperative immersion in blood or bone marrow aspirates) for cranial defect reconstruction during craniosynostosis correction surgeries in eight children. After 12 months, two patients exhibited noticeable voids without bone regeneration, while significant degradation of PCL was also noticed (see Figure 4I). In comparison to reconstruction using autologous particulate bone grafts, PCL implants resulted in inferior bone regeneration after 9 months post-operatively. The authors suggested that the non-bioactive PCL implant occupied the defective area and hindered intrinsic tissue regeneration.

As concluded from the case reports, 3D-printed PCL-based implants have demonstrated significant value in burr hole reconstruction. However, cranioplasty for large-sized defects has shown mixed results. A successful treatment evidenced by robust bone formation likely requires angiogenic and osteogenic bioactive molecules (e.g., additives like β-TCP, cells, and cytokines) and sufficient blood supply from surrounding soft tissue, as demonstrated in a recent publication [108]. However, it shall also be noted that PCL degrades slowly, and the residual material may hinder the growth of new bone tissue [107]. It is also unclear whether the PCL matrix, which is known for low stiffness and low strength, may protect the brain from accidental mechanical impacts [109]. Last but not least, the acidic biodegradation products may potentially jeopardise the bone healing process, which favours a neutral to the slightly basal environment [110].

In summary, this section has reviewed three types of implants that are specifically designed for regenerative cranioplasty. As listed in Table 2, certain implants have shown promising results, contributing to uneventful healing and progressive bone regeneration. Conversely, other implants have exhibited a higher incidence of complications, indicating the need for future research to find solutions.

## 4. Perspectives on Enhancing the Regenerative Cranioplasty Implants

After examining the clinical trials of various regenerative cranioplasty implants, this section explores the necessary steps in the research and development of implants to ensure safer and more effective healing. While some perspectives are derived from reported clinical outcomes for some known concerns, others, although less discussed in existing literature, are considered clinically significant for cranium regeneration.

### 4.1. Enhancing the Osteogenic Potential on the Scalp Side

Among the in vivo studies and clinical case reports of spontaneous cranium regeneration, a common feature is that new bones were predominantly formed on the dural side of the implant, whereas bone formation at the scalp/pericranium side was less notable. Moreover, the regenerated bone is typically thinner than the natural cranial bone, presenting a concavity on the scalp side; see Figure 1(A-IV,C-IV). While it is possible that the time reporting the spontaneous regeneration was too early to witness a full-thickness cranium regeneration, it remained intriguing as to why the bone formation at the scalp side of the implant is less significant and whether the regeneration can be stimulated at this side to achieve a full-thickness regeneration of cranium more efficiently and homogeneously.

The discrepancy in bone formation on the two sides of the cranium is attributed to the different osteogenic potentials of the dura and pericranium, with the latter being the bottom component of the scalp that directly contacts the cranium. The growth and regeneration of the cranium are dependent on the recruitment of osteoprogenitor cells and blood supply from three sources: dura, pericranium, and diploë, the vascularised cancellous bone of the cranium [78]. Despite being non-osseous tissue, both pericranium and dura with structural and functional integrity were considered prerequisites for spontaneous cranium regeneration. Compared to pericranium, dura was proven in animal studies to have greater significance in supporting cranial regeneration throughout the defect [118,119,120], owing to a more abundant supply of blood and osteoprogenitor cells to the defective site [78], whereas pericranium contributes to bone regeneration at a close adjacency [119]. To this end, strategies to improve the availability of osteogenic cells or blood supply near the pericranium may compensate for the inferior osteogenic potential of the pericranium and promote local bone healing.

To instantly improve the availability of cells at the pericranium site, a straightforward approach is the transplantation of autologous cells at the defective sites. Previous in vivo studies have demonstrated that when seeded onto a scaffold, a huge variety of cells, including the endothelial (progenitor) cells forming blood vessels [121,122], and pluripotent mesenchymal stem cells sourced from bone marrow [123,124], fat tissue [125,126], umbilical cord [127,128], dental pulp [129], urine [130], or derived from human induced pluripotent stem cells [131,132], can induce the regeneration of cranium more effectively than cell-free counterparts. Encouraged by these cases, clinical trials were carried out to evaluate the pluripotent stem cells, either sourced from fat [133,134,135] or bone marrow [136], for their capacity to induce the regeneration of the cranium. The short-term follow-ups consistently reported uneventful healing with increased bone density inside the implants and in the defective areas. However, regarding the long-term follow-up, unsatisfactory outcomes were reported by two groups. Thesleff et al. noticed poor ossification and graft resorption in 3 out of 5 patients receiving adipose-derived stem cells/beta-tricalcium phosphate bone grafts [134], while Morrison et al. reported graft resorption in all three cases of cranioplasty using sandwich constructs comprised of poly(L-lactide-co-glycolide) (PLGA) sheet—mesenchymal stem cell/ChronOS ceramic granules mixture—PLGA sheet [136]. In both reports, the authors confirmed the safety and effectiveness of stem cells, as eventless healing accompanied by increased bone density was noticed in the early stages of therapies, while the eventual failures were attributed to mechanical instability at the implant sites (see Section 4.4). While the stem cells proved to be promising in regenerative cranioplasty, care must also be taken to ensure good viability of the transplanted cells, as they hardly survive beyond 3 weeks after transplantation [137]. Strategies like improving the local oxygen supply may enhance the survival of transplanted cells and increase the successful rate of healing [138,139].

**Figure 5:**
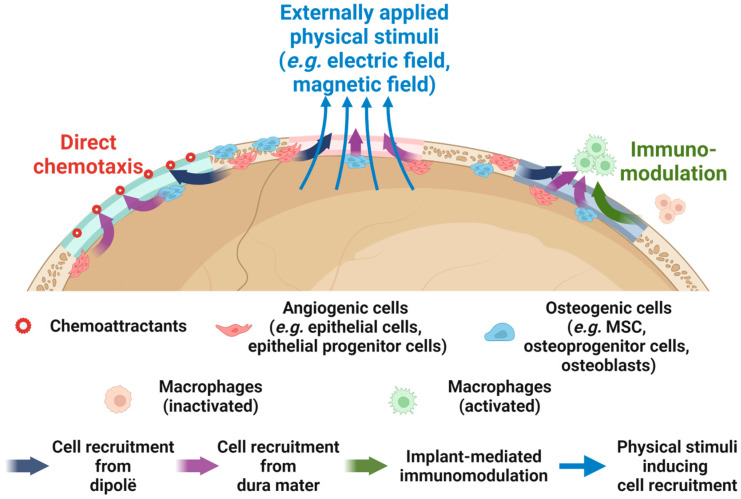
Schemes of cell recruitment are mediated by a regenerative cranioplasty implant via different mechanisms, including direct chemotaxis, application of physical stimuli, and immunomodulation. MSC = mesenchymal stem cells. Image created with BioRender (www.biorender.com, accessed on 31 January 2024) with permission to publish.

Alternatively, the availability of cells on the scalp side can be improved through the in situ recruitment of osteogenic or angiogenic cells. In comparison to cell transplantation, in situ cell recruitment avoids the need for an invasive approach to harvest cells, reduces donor site morbidity, and eliminates the time-consuming processes of cell isolation and in vitro expansion, making it more favourable for clinical practice [140]. As listed in Table 3, enhancing the in situ migration of pluripotent stem cells and endothelial (progenitor) cells has been proven effective in inducing cranial bone regeneration. Many of these studies relied on immunomodulation, where macrophages polarized toward the anti-inflammatory/pro-regenerative M2 phenotype, thereby promoting the recruitment of endogenous stem cells for tissue regeneration. Xu et al. also reported that a moderate pro-inflammatory microenvironment in the early stages after scaffold implantation contributed to stem cell recruitment. Alternatively, cell recruitment could be promoted by local administration [141,142] or in situ expression [143] of chemoattractants that are responsible for cell homing, as well as by physical cues (e.g., magnetic field) [144]; see Figure 5 for a schematic illustration.

A protein proven to be both chemoattractive to bone-forming cells and pivotal to the development and regeneration of cranium is bone morphogenic protein-2 (BMP-2) [46]. Early studies by Lind et al. and Fiedler et al. both reported that BMP-2 was able to induce the chemotactic migration of both differentiated osteoblasts and progenitor cells in a dose-dependent manner, with the optimal concentration in cell culture medium being 1 ng/mL [145,146]. In combination with stromal cell-derived factor-1 protein (SDF-1), a molecule that plays a decisive role in the early stage of bone defect healing to recruit MSCs, BMP-2 was found to reinforce cell recruitment and, more importantly, effectively induce the osteogenic differentiation of stem cells, thereby leading to significant regeneration of rat cranial defects [147,148]. Since the early 2000s, surgeons have been applying BMP-2 in regenerative cranioplasty. Arnander et al. reported a case of frontal bone reconstruction using an ectopically ossified bone graft, which was obtained by administration of BMP-2 over the latissimus dorsal muscle that was embedded in a customised mould implanted at the back [149]. Later, Kohan et al. reported 8 cases of regenerative cranioplasty using a collagen sponge absorbed with BMP-2 and sandwiched by poly-(D, L)-lactide resorbable meshes. A dramatically higher rate of bone regeneration at POY 1 (~85%) was noticed compared to non-resorbable materials (i.e., titanium, PEEK), where virtually no bone regenerated, whereas the perioperative complication rate for BMP-2-containing constructs (14%) was also significantly lower than titanium (64%), cryopreserved cranium (50%) and PEEK (38%), but slightly inferior to autologous bone (5%) [150]. These reports demonstrate the promising role of BMP-2 in regenerative cranioplasty. Future studies are required to understand the optimal dosage and, more importantly, achieve a controlled release of BMP-2 at the defect site to avoid undesired effects such as inflammation, hematoma, and bone cysts [151].

Pre-vascularised tissue engineering constructs, which contain blood vessel networks formed ex situ before implantation, have also been purposed for cranial bone defect repair. It is assumed that the pre-developed vascular network will quickly anastomose with the host vasculature, thereby supplying oxygen and nutrients throughout the tissue engineering construct to facilitate the bone regeneration process [152]. Nonetheless, Roux et al. only noticed an insignificantly greater bone formation after implantation of fibrin-based scaffolds containing pre-vascularised HUVEC/MSC coculture spheroids, compared to either cell-free or MSC-only scaffolds [153]. The authors later prepared pre-vascularised tissue engineering constructs using HA, fibrin, and spheroids co-culturing MSC and endothelial cells derived from induced pluripotent stem cells [154]. While the pre-vascularised construct outperformed the MSC-only constructs and resulted in a higher bone mineral density and vascularisation at the rat cranial defects, the area of mature bone in the histological images was marginally higher and showed no statistical significance. More effort is required to verify the effectiveness of pre-vascularised tissue engineering constructs in cranial defect regeneration.

**Table 3:** Factors applied to promote cell recruitment and enhance cranial bone regeneration based on in vitro and in vivo studies.

Factors	Type of Factors	Carrier of the Factors	Animal Species And Size of Cranial Defects	Impact on Cell Recruitment	Ref.
IL-4	Biological	Electrospun PLGA/HA scaffolds loaded with IL-4 and coated with carboxymethyl chitosan–collagen–hydrogel	SD rats D = 5 mm	The hydrogel coating impeded the IL-4 release in an early stage (Day 1–3) to maintain a moderate pro-inflammatory response that recruits BMSC. Afterwards, the hydrogel degraded and released trapped IL-4 to induce an anti-inflammatory response to upregulate cranium regeneration	[155]
BML-284, carboxymethyl chitosan	Biological	β-TCP with PDA coating	Rats D = 5 mm	Surface functionalisation with BML-284 and carboxymethyl chitosan promoted the M2 (anti-inflammatory) polarisation of macrophages and facilitated endogenous MSC recruitment to support cranium regeneration	[156]
HyA-DA	Biological	Collagen I/HA composite hydrogel	NZ rabbits D = 10 mm	The presence of HyA-DA at the Collagen I/HA interface activated the M2 polarisation of macrophages to induce the endogenous MSC recruitment and subsequent cranial defect repair	[157]
LepR-a	Biological	PLA with PDA coating and BMP-2 loaded hollow MnO_2_ nanoparticles	C57BL/6 mice D = 4 mm	Surface functionalisation with LepR, a surface marker for >90% of Prx1^+^ SSC, recruited SSC (in vitro and in vivo) through antibody-antigen reaction and contributed to enhanced cranium regeneration	[158]
SDF-1	Biological	CSO/H NPs	Nude mice subcutaneous	SDF-1 released from nanoparticles induced in vitro and in vivo MSC recruitment, while BMP-2 (released subsequently) enhanced osteogenic differentiation of stem cells.	[148]
BMP-2	Biological	Phosphate buffered saline (for injection) or collagen scaffold	C57BL/6 mice D = 5 mm	In vitro administration of BMP-2 enhanced the chemotactic migration of osteoblasts by 170–300%; Sequential release of SDF-1 (from scaffold) followed by BMP-2 (injection) dramatically enhanced the recruitment and osteogenic differentiation of BMSCs, leading to enhanced calvaria defect healing	[146,147]
FGF-2	Biological	BMP-2 loaded HA/collagen scaffold with polyelectrolyte multilayer coating	Mice D = 3.5 mm	Sequential release of FGF-2 (from coating) followed by BMP-2 (from scaffold) greatly enhanced the recruitment of Sca-1+ progenitor cells and subsequent osteogenesis at the calvaria defects of mice	[159]
PDGF-B	Biological	Recombinant adenoviruses loaded in mesoporous glass–silk scaffolds	BALB/c mice 5 × 5 mm^2^	Release of adenoviruses from the scaffold was able to infect MSC, PDLSC, and DPSC, leading to enhanced production of PDGF-B that subsequently improved the migration of these (undifferentiated) cells to support calvaria defect healing.	[160]
PFS (CPFSSTKT-NH2) peptide	Biological	SCP/SF scaffold	SD rats D = 5 mm	Functionalisation of SCP/SF with PFS peptide, a peptide with stem cell-homing ability, enhanced both in vitro and in vivo recruitment of MSC to promote cranium regeneration	[141]
Ground autologous bone	Biological	Bioprinted alginate–gelatin hydrogel scaffold	Beagle dogs 20 × 20 mm^2^	Implantation of scaffolds containing ground autologous bone and transplanted BMSC into cranium defects of rats upregulated the expression of SDF-1 and consequently enhanced in situ recruitment of CD90+/CD105+ BMSC to support cranium regeneration	[143]
Lithium-doped BG	Chemical	PLGA	C57BL/6 mice D = 4 mm	Incorporation of Li in BG enhanced in vitro migration of BMSC under low/high glucose environment and contributed to superior regeneration of calvaria defect in mice with diabetes	[161]
Lanthanum-doped BG	Chemical	Chitosan	SD rats D = 5 mm	Increased loading of La-BG improved in vitro recruitment and subsequent expression of angiogenesis-related genes of HUVEC to improve cranium regeneration	[162]
Calcium ion (from CaSO_4_)	Chemical	Agarose/chitosan scaffold	BALB/c mice D = 5 mm	Calcium ions released from the scaffold enhanced in situ recruitment of Osx+ osteoprogenitor cells at the mice calvaria defect and subsequently resulted in more pronounced bone regeneration	[163]
Piezoelectric stimulus	Physical	PHA and PBT in CG	Rats Unknown size	PHA/CG/5%PBT hydrogel most effectively induced migration and M2 polarisation of RAW 264.7 cells (murine macrophages), which subsequently enhanced the in vitro recruitment of MC3T3-E1 (murine pre-osteoblasts) and HUVEC, and facilitated cranium regeneration	[164]
SrFe_12_O_19_ nanoparticles	Physical	Lanthanum-doped HA/CS scaffold	SD rats D = 5 mm	The incorporation of magnetic SrFe_12_O_19_ nanoparticles induced an incorporated magnetic field and promoted the recruitment of MSC to enhance cranium regeneration	[144]

Abbreviations: IL-4 = interleukin 4; PLGA = poly(lactic-co-glycolic acid); SD rats = Sprague–Dawley rats; D = diameter; BMSC = bone marrow-derived stem cells or bone marrow stromal cells; BML-284 = Wnt signalling activator I; HyA-DA = dopamine-modified hyaluronic acid; HA = hydroxyapatite; NZ rabbits = New Zealand rabbits; MSC = mesenchymal stem cells; LepR-a = Lep receptor antibody; SDF-1: Stromal cell derived factor-1; CSO/H NPs = chitosan oligosaccharide/heparin nanoparticles; PLA = polylactic acid; β-TCP = β-tricalcium phosphate; PDA = polydopamine; SSC = skeletal stem cells; BMP-2 = bone morphogenetic protein 2; FGF-2 = fibroblast growth factor 2; PDGF-B = platelet-derived growth factor-B; PDLSC = peridontal ligament stem cells; DPSC = dental pulp stem cells; BG = bioactive glass; HUVEC = human umbilical vascular endothelial cells; PHA = PDA-treated hydroxyapatite; PBT = PDA-treated BaTiO3; CG = chitosan/gelatin composites; SCP = silicon calcium phosphate; SF = silk fibroin; CS = chitosan.

### 4.2. Proper Management of Surrounding Soft Tissue

**Figure 6:**
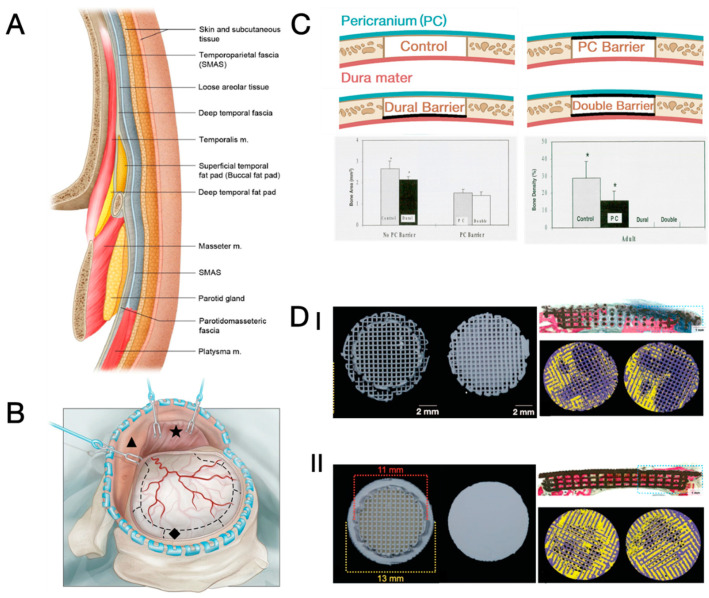
(**A**) Schematic view of anatomy in calvarial and temporal regions showing the multiple layers of soft tissues [165]; (**B**) illustration of craniectomy, during which the scalp (▲), temporalis (★), and dura mater (◆) are dissected from the cranium [174,175]; (**C**) schematic view and key results of a study demonstrating the contribution of dura mater and pericranium to the bony regeneration in sub-critical cranial defects of rats. * denotes statistical significance (*p* < 0.05) [118]. (**D**) Pre-implantation photographs, post-surgical Stevenel’s blue and Van Gieson’s picro fuchsin differential tissue staining (SVG staining, blue = soft tissue, red = mineralised bone, black = scaffold), and CT reconstructions (yellow = new bone) at POW 8 for porous β-TCP scaffold with (I) porous cap and (II) solid cap [173]. Both materials were implanted into cranial defects of rabbits, where the top of the scaffold directly interacts with Galea aponeurotica. Reprinted from Ref. [174]; Reprinted with permission from Ref. [165]. Copyright 2024, Thieme; Refs. [118,173]. Copyright 2024, Wolters Kluwer Health, Inc.

While many works focused on achieving integration between the cranioplasty implant and surrounding cranium to ensure good implant stability and cell recruitment from dipolë to the defect area, few works focused on the interaction of the cranioplasty implant with the surrounding soft tissues, i.e., dura mater, pericranium, and coarse connective tissue beneath the temporalis (see Figure 6A) [165]. Compared to the dipolë, these soft tissues typically have a larger area in direct contact with a cranioplasty implant, and recent studies revealed the significance of these soft tissues in the healing of cranial defects. It is known that pericranium, dura mater, and temporalis possess osteogenic potential [78,166], and cases of spontaneous cranium regeneration similarly emphasise the potential contribution of the intact or carefully reconstructed dura mater and pericranium to the re-ossification at the defective sites [47,49,54]. An intimate integration of a healthy, vascularised scalp with a cranioplasty implant also contributes to greater implant stability and a lower risk of infection [20,106,167,168,169], while close contact between the dura mater and implant is vital to minimise the interval of dead space and subsequent fluid collection [170]. Moreover, failure of stable integration between temporalis and cranioplasty implant, especially at the rim region, leads to repetitive friction that eventually causes fluid collection, dehiscence, and injury of soft tissue [18,19,85]. These findings suggest that the soft tissues, either damaged and removed after trauma or dissected away from the cranium during craniectomy (see Figure 6B), significantly contribute to an uneventful and effective regeneration of the cranium.

The significance of these surrounding soft tissues suggests that the interaction of a regenerative cranioplasty implant and surrounding soft tissue may differ from the conventional understanding of guided bone repair, where the soft tissue shall be segregated from the bone tissue engineering construct, as the invasion of fast-growing soft tissue would impede bone regeneration otherwise [171,172]. An early study revealed that a barrier between either the pericranium or dura mater and a defective area hampered the regeneration of the cranium and the viability of transplanted bone grafts (Figure 6C) [118,119]. In cases where pericranium is damaged or removed, however, the direct exposure of Galea aponeurotica results in undesired fibrous tissue ingrowth and hampered cranial bone regeneration (Figure 6D) [173]. Considering the great complexity of the spatial distributions of the soft tissues over the implant as well as the unique impacts of each soft tissue on cranium regeneration, a universal principle for the interaction between a regenerative cranioplasty implant and the surrounding soft tissue is unlikely to be established.

The enormous potential of soft tissue in facilitating cranium regeneration should be fully exploited in future research. To this end, an imminent issue is to develop a comprehensive understanding of how each type of soft tissue would affect cranium regeneration. While the roles of dura mater and pericranium were previously reported, more effort is required to elucidate the influence of loose connective tissue, Galea aponeurotica, and temporalis, all on the scalp side. This understanding will then be the reference to determine whether a specific soft tissue shall be integrated with or separated from the regenerative cranioplasty implants to ensure robust bone regeneration. Ultimately, engineers must take responsibility for properly designing and processing biomaterials so that cranioplasty implants possess differently processed/designed regions to induce anticipated responses (e.g., repellent or adhesion) of specific soft tissues and to support the regeneration of the cranium.

### 4.3. Endochondral Ossification as an Alternative Ossification Mode

**Figure 7:**
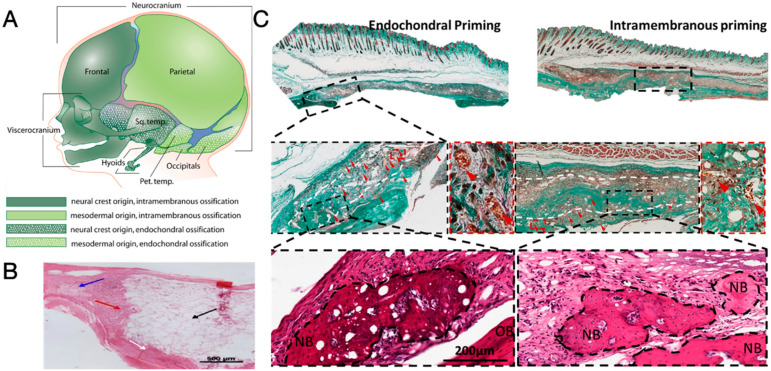
(**A**) Diagram showing the origin and mode of ossification for different regions of craniofacial bones [184]; (**B**) hematoxylin and eosin staining section of a collagen–calcium phosphate scaffold seeded with bone marrow-derived mesenchymal stem cells and implanted into the cranium of Wistar rats for 8 weeks. The blue arrow highlights mineralised new bone, the red arrow highlights dense bone at the periphery of the scaffold, and the loosely packed tissue highlighted by the black arrow is a necrotic region in the scaffold centre [185]. (**C**) Histological sections (Masson trichrome and hematoxylin and eosin) of cranial defects regenerated by polycaprolactone microfibrous scaffold seeded with mesenchymal stem cells and primed for endochondral ossification (left) and intramembranous ossification (right). Red arrows highlight bone vessels. OB = original bones; NB = new bones [186]. Reprinted with permission from Refs. [184,185]. Copyright 2024, Elsevier; Reprinted from Ref. [186].

As thoroughly discussed by Ko and Sumner, the regeneration of the cranium undergoes a process that assimilates intramembranous ossification (IMO) [43], as most of the cranial bones are formed by the IMO process (Figure 7A) [42], and lack of strain at cranial bone defects does not favour the formation of cartilaginous callus, a typical sign of endochondral ossification (ECO) and bone regeneration [43,176]. Consequently, most tissue engineering constructs intended for cranial defect healing were designed to induce IMO, where mesenchymal stem cells differentiate into osteoprogenitor cells and then osteoblasts. Nevertheless, many of these tissue engineering constructs failed to ensure good vascularization at the regeneration site. Ultimately, poor vascularity, especially at the core of tissue engineering constructs, led to cell necrosis and failed bone regeneration, as depicted in Figure 7B [177].

In contrast to the IMO, the ECO is an ossification process that predominantly takes place in long bones [41]. During this process, a temporal cartilaginous matrix with MSC condensation is first formed, giving rise to chondrocytes that subsequently undergo hypertrophic differentiation [41,178]. The hypertrophic chondrocytes within the matrix then produce vascular endothelial growth factor (VEGF) to promote blood vessel invasion into the matrix, thereby supporting the subsequent ossification activities [179,180]. Researchers considered the use of ECO-inducing tissue engineering constructs to guide the regeneration of highly vascularized bone tissue in the cranium. The contribution of ECO to the regeneration of the cranium was demonstrated by Kanou et al., who transplanted the periosteum stripped from the tibia and cranium to heal cranial defects. The tibial periosteum induced cranial regeneration via both ECO and IMO after transplantation, leading to superior defect healing compared to the cranial periosteum, which induced a smaller area of bone formation via IMO only [181,182]. Following in vitro chondrogenic-hypertrophic priming, an MSC-laden hydrogel induced for ECO was also found to facilitate cranial defect healing [178]. It was later reported that, compared to tissue engineering constructs primed for IMO, those primed for ECO, i.e., induction of chondrogenic differentiation followed by hypertrophy induction, resulted in more significant regeneration of defective cranium, as evidenced by greater areas of newly formed bones and a greater number of blood vessels within the cranial defects (Figure 7C) [177]. While these studies demonstrated the potential of ECO in cranial defect healing, a premise for success is that the innate angiogenesis function of the patient is not jeopardised; otherwise, the presence of hypertrophic chondrocytes is prolonged, and the ossification stage is delayed [183]. Future studies are required to investigate the time efficiency of ECO-mediated cranium regeneration in other animal models and to characterise any structural and compositional differences in the bones regenerated via ECO and IMO.

### 4.4. Consideration of the Local Mechanical Environment

While the cranium is generally considered a non-load-bearing skeletal structure [187], an inlay cranioplasty implant in close contact with the dura mater experiences mechanical loading from cyclic brain pulsation, which is known to disrupt the setting of bone cement and ultimately leads to premature cement fragmentation [188]. A previous study suggests that resorbable polymeric meshes may dampen the mechanical impact of the pulsation, protecting resorbable CaP cement seeded with stem cells from breaking down [189]. Although short-term follow-up results were satisfying, long-term follow-ups showed that the construct ultimately became loosened, with degenerated, discontinuous bone tissue distributed within [134,136]. This contrasts with cases where a rigid titanium mesh was used as the buffering material, resulting in successful ossification and osteoconduction [133,134]. It is recognised that mechanical stimuli hasten the loss of strength and stiffness in resorbable polymers [190,191]. As the stiffness and strength of the polymeric mesh deteriorate, the cranioplasty implant becomes more susceptible to micromotion induced by cerebral pulsation [134]. Consequently, the regenerated bone at the implant-cranium interface is ruptured, and the implant becomes loosened. Moreover, the foreign body reaction (fibrous encapsulation) is exacerbated following implant loosening, and cell-mediated degradation becomes intensified, accelerating the resorption of the implant and causing implant failure [192].

What we learned from cases of implant loosening and catastrophic fragmentation is that the role of inherent mechanical stimuli in the fate of regenerative cranioplasty implants, long neglected in previous studies, demands systematic investigation before any clinical trial takes place. Fortunately, the characterisation of cerebral pulsation is available through various approaches, such as invasive intracranial pressure measurement, transcranial Doppler ultrasound, and magnetic resonance imaging, making it possible to establish a dynamic mechanical loading regime for in vitro testing [193]. To better simulate the post-implantation condition, the cyclic mechanical load simulating cerebral pulsations shall be applied, in addition to testing conditions where the temperature and pH of the surrounding aqueous phase assimilate those in the human body.

While in vitro biomechanical tests are valuable for quantitatively investigating the impact of cerebral pulsations on the fate of cranioplasty implants, an in vivo study best mimics the actual condition of clinical application, where a dynamic biological environment and inherent cerebral pulsation both affect the degradation behaviour of implants. The animal model shall be carefully determined so that the cerebral pulsation recapitulates that of human beings. As dural pulsations are synchronic with heartbeats, large animals (sheep, pigs), whose heart rates are closer to those of human beings, are superior to rats and rabbits, which bear much higher heart rates, and shall be selected where funding allows [194]. On the basis of establishing a reliable testing protocol, material scientists bear the responsibility of developing novel materials that are stiff and strong enough to withstand the impact of physiological mechanical loads while maintaining good biodegradability upon implantation.

## 5. Conclusions

This work reviews the current advances in the research, development, and clinical application of biomaterials intended for regenerative cranioplasty. Despite a long-held belief that the cranium does not regenerate after the age of 2, there is an increasing number of cases discussing the spontaneous regeneration and ossification of the defective cranium in adolescents and adults. The clinical observation, along with the finding of multipotent cells residing in the cranial suture and signs of upregulated osteogenic activities upon cranial defects, underlines the capacity of cranium regeneration beyond the age of 2 and calls for further research to investigate the underlying mechanisms and, hopefully, how tissue engineering may contribute to clinical healing of cranial defects.

Currently, three types of biomaterials have gone through clinical translation for regenerative cranioplasty. The CaP/Ti composites have been applied in nearly 2000 clinical cases, and the material presents a low complication rate, osteoinductivity, and, more importantly, a coupled progress between implant degradation and bone regeneration. Mineralised collagen is considered highly biomimetic to native bone regarding its chemical composition and nanostructure. However, retrospective studies reported a considerable ratio of complications in paediatric cranioplasty, with implant fragmentation and loosening being the most critical conditions. Three-dimensional-printed PCL and β-TCP/PCL composites have shown promising clinical outcomes in both burr hole reconstruction and large-sized cranioplasty, whereas the low bioactivity and slow degradation process of PCL are considered by some clinicians to impede the healing progress. By incorporating autologous cells and cytokines during implantation surgeries, bone regeneration can be facilitated, and the success rate of PCL-based cranioplasty implants may be improved.

Based on the theoretical understanding of cranial regeneration as well as the issues reported from clinical trials, there are four potential directions to enhance the safety and effectiveness of regenerative cranioplasty implants. Seeing the manner of a less prominent bone formation at the scalp/pericranial side, it is considered that the availability and osteogenic potential of cells at the scalp/pericranial side of implants shall first be enhanced. Considering the contribution of the scalp and dura mater in the regeneration of the cranium, a proper interaction between the surrounding soft tissues and the implants shall also be established. Priming the tissue engineering constructs for endochondral ossification is another appealing option, as it enhances the local vascularity and contributes significantly to bone development. Finally, the role of the inherent mechanical environment presented at the cranial defects shall not be neglected.

The development of regenerative cranioplasty implants is still in an early stage. While positive clinical outcomes have been documented for some implants, the follow-up period is yet too short to comprehensively evaluate the safety and effectiveness of healing, calling for multi-centre clinical trials with larger cohorts and longer follow-ups in the future. Continuous cross-disciplinary research in developmental biology, regenerative medicine, biomaterials, and biofabrication is also necessary for the development of safer and more effective implants for uneventful, efficient cranium regeneration.

## Figures and Tables

**Table 2:** Comparison of three types of regenerative cranioplasty biomaterials.

Category	Biodegradability	Mechanical Properties of Major Components	Bone-Bonding and Bone Regeneration	Highlights of Reviewed Clinical Studies
CaP/Ti	Partly biodegradable	Ti: ~900 MPa (UTS) [111] 105–125 GPa (YM)[111] CaP (Monetite *, dense): ~445 MPa (UTS) [112] ~377.5 GPa (YM) [112]	Yes	The low complication rate in general Evidence of bone formation over and between the formulated CaP tiles Not fully biodegradable No study on impact over residual titanium mesh
Mineralised collagen	Fully biodegradable	Collagen (bulk): 2–90 MPa (UTS) [92,113] <2 GPa (YM) [114] HA: 308–509 MPa (CS) [115] 42.2–81.4 GPa (CM) [115]	Yes	Mainly applied in paediatric cranioplasties Moderate incidence rate of hydrops, implant fragmentation, and implant loosening due to premature resorption
Three-dimensional-printed PCL and β-TCP/PCL composites	Fully biodegradable	PCL: ~28.7 MPa (UTS) [109] 0.25 GPa (YM-Tension)[109] β-TCP: 1–10 GPa (UTS-Theoretical) [116] ~110 GPa (YM) [116]	No (PCL) Yes (β-TCP/PCL)	Low complication rates in general Increased radiodensity at the defective sites after implantation, likely due to soft tissue formation Coating with blood/bone marrow aspirate and coverage of pedicled flap may enhance clinical outcome Slow degradation may hinder bone regeneration
Human calvaria	-	43–79 MPa (UTS) [117] 11.7–15.0 GPa (YM) [117]	-	-

* Monetite is the major (>85%) component of the CaP in OssDsign implant.
